# Clinical Outcomes in Patients With T4b Esophageal Squamous Cell Carcinoma: A 10‐Year Single Institution Experience

**DOI:** 10.1002/kjm2.70116

**Published:** 2025-09-25

**Authors:** Yi‐Hsun Chen, Wen‐Hung Hsu, I‐Chen Wu, Pen‐Tzu Fang, Song‐Wei Wang, Chao‐Chin Hsu, Yao‐Kuang Wang

**Affiliations:** ^1^ Division of Gastroenterology, Department of Internal Medicine, Kaohsiung Medical University Hospital Kaohsiung Medical University Kaohsiung Taiwan; ^2^ Graduate Institute of Clinical Medicine, College of Medicine Kaohsiung Medical University Kaohsiung Taiwan; ^3^ School of Post Baccalaureate Medicine, College of Medicine Kaohsiung Medical University Kaohsiung Taiwan; ^4^ College of Medicine Kaohsiung Medical University Kaohsiung Taiwan; ^5^ Biomedical Artificial Intelligence Academy Kaohsiung Medical University Kaohsiung Taiwan; ^6^ Department of Radiation Oncology Kaohsiung Medical University Hospital Kaohsiung Taiwan; ^7^ Department of Radiation Oncology, Faculty of Medicine Kaohsiung Medical University Kaohsiung Taiwan

**Keywords:** chemoradiotherapy, esophageal fistula, esophageal squamous cell carcinoma, T4b

## Abstract

Esophageal squamous cell carcinoma (ESCC) with adjacent structure invasion (T4b), affecting areas such as the aorta, vertebral body, or trachea, is associated with a poor prognosis, and the optimal treatment strategy remains unclear. While chemotherapy is considered standard, radiation therapy is often avoided due to the risk of severe complications, including tracheoesophageal fistula. This 10‐year cohort study investigated clinical outcomes and prognostic factors in patients with T4b ESCC. From October 2011 to May 2022, 471 ESCC patients were diagnosed at our institution, of whom 130 (27%) had T4b disease. First‐line treatments included definitive chemoradiotherapy (*n* = 82), chemotherapy alone (*n* = 15), radiotherapy alone (*n* = 5), immunotherapy clinical trials (*n* = 7), palliative surgery (*n* = 2) and best supportive care (*n* = 19). Patients treated with definitive chemoradiotherapy demonstrated significantly longer overall survival compared with those receiving monotherapy. The mean survival was 24.2 months in the chemoradiotherapy group, versus 4.2 months with chemotherapy alone and 8.5 months with radiotherapy alone (*p* < 0.001). Esophageal fistula developed in 22 patients (16.9%), with 5 cases identified at diagnosis and 17 occurring during follow‐up. Chemotherapy alone was associated with a significantly higher risk of fistula formation compared with chemoradiotherapy (adjusted hazard ratio = 11.22, *p* = 0.01). The presence of a fistula was correlated with worse survival outcomes (median survival of 8.9 months versus 12.2 months, *p* = 0.03). These findings suggest that definitive chemoradiotherapy may enhance survival in T4b ESCC patients.

## 1 Introduction

Esophageal squamous cell carcinoma (ESCC) accounts for 85% of esophageal cancer globally, with an estimated 512,500 new cases in 2020. High prevalence rates are observed in Eastern and Southern Africa and Eastern Asia [1]. The estimated number of new cases and deaths from ESCC will be 806,000 and 748,000 respectively in 2040 [2], highlighting the urgent need for improved treatment strategies. The 5‐year survival rate remains low (10%–30%) in most countries [3]. In Taiwan, esophageal cancer ranks ninth in incidence and fifth in mortality in the general population, disproportionately affecting middle‐aged men with approximately two‐thirds diagnosed at advanced stages (III/IV) and carrying significant socioeconomic consequences [4].

T4b ESCC represents tumors invading adjacent organs such as the airway, aorta, or vertebral body [5], represents a particularly challenging subset of the disease. The prevalence of T4b ESCC is estimated at approximately 10%–12% [6, 7, 8]. While definitive chemoradiotherapy (dCRT) is an established treatment option [9], the prognosis remains poor, with median overall survival reported at 1–1.5 years [8, 10]. Esophageal fistula is a major complication in patients with T4b ESCC. The presence of esophageal fistula is associated with treatment failure and reduced survival [8, 11, 12], further complicating management.

This study aimed to retrospectively analyze clinical outcomes and identify risk factors associated with esophageal fistula formation in a 10‐year single‐center cohort of patients with T4b ESCC, addressing a critical unmet need in the management of this aggressive disease.

## 2 Materials and Methods

### 2.1 Patients and Ethical Approval Statement

This prospective cohort study enrolled patients diagnosed with esophageal squamous cell carcinoma (ESCC) at a single medical center in Taiwan between October 2011 and May 2022. Patients with non‐ESCC cancers or more than one primary cancer were excluded. Data analysis was restricted to patients with T4b ESCC and analyzed retrospectively. This study was approved by the Institutional Review Board of Kaohsiung Medical University Hospital (KMUHIRB‐E(I)‐20240427).

All patients received a comprehensive assessment including laboratory tests, imaging studies, such as chest X‐ray, chest/abdominal computed tomography (CT), positron emission tomography (PET) and esophagogastroduodenoscopy (EGD). Baseline body mass index (BMI) and Eastern Cooperative Oncology Group (ECOG) performance status were recorded prior to treatment.

### 2.2 Treatment Options for T4b ESCC Patients

Treatment regimens were determined collaboratively by the ESCC consortium and individual patient discussions. First‐line therapy consisted of concurrent chemoradiotherapy. Chemotherapy comprised cisplatin (75 mg/m^2^) administered intravenously on day 1, followed by a continuous intravenous infusion of 5‐fluorouracil (5‐FU) (1000 mg/m^2^) from days 1–4, repeated every 4 weeks. Carboplatin (area under the curve = 5) replaced cisplatin if creatinine clearance was < 50 mL/min. Radiation therapy with a total 50.4 Gray in 25–28 fractions was initiated within 1 month of chemotherapy [13, 14]. Chemotherapy was temporarily withheld for grade 3 or higher toxicity and resumed once toxicity improved to grade 2 or less. Selected patients participated in immunotherapy clinical trials. Patients who chose not to pursue further treatment received the best supportive care through hospice consultation.

### 2.3 Esophageal Fistula Assessment

Esophagogastroduodenoscopy (EGD) and bronchoscopy were used to assess the esophageal fistula, while tracheal invasion by the esophageal tumor was assessed using the cough test and endobronchial ultrasonography. Esophageal fistula was assessed every 3 months using EGD and chest CT; the interval was shortened if the patient experienced persistent post‐prandial cough or recurrent pneumonia.

### 2.4 Statistical Analysis

Categorical variables were presented as counts and percentages, while continuous variables were expressed as means with standard errors, with differences in continuous variables assessed using the Kruskal–Wallis test and categorical variables analyzed with Fisher's exact test. Cox proportional hazards regression was employed to evaluate overall survival and risk factors for esophageal fistula. Survival outcomes were estimated with the Kaplan–Meier method and compared using the log‐rank test. All analyses were performed using STATA (version 15.0). A two‐tailed *p* < 0.05 was considered statistically significant.

## 3 Results

**FIGURE 1:**
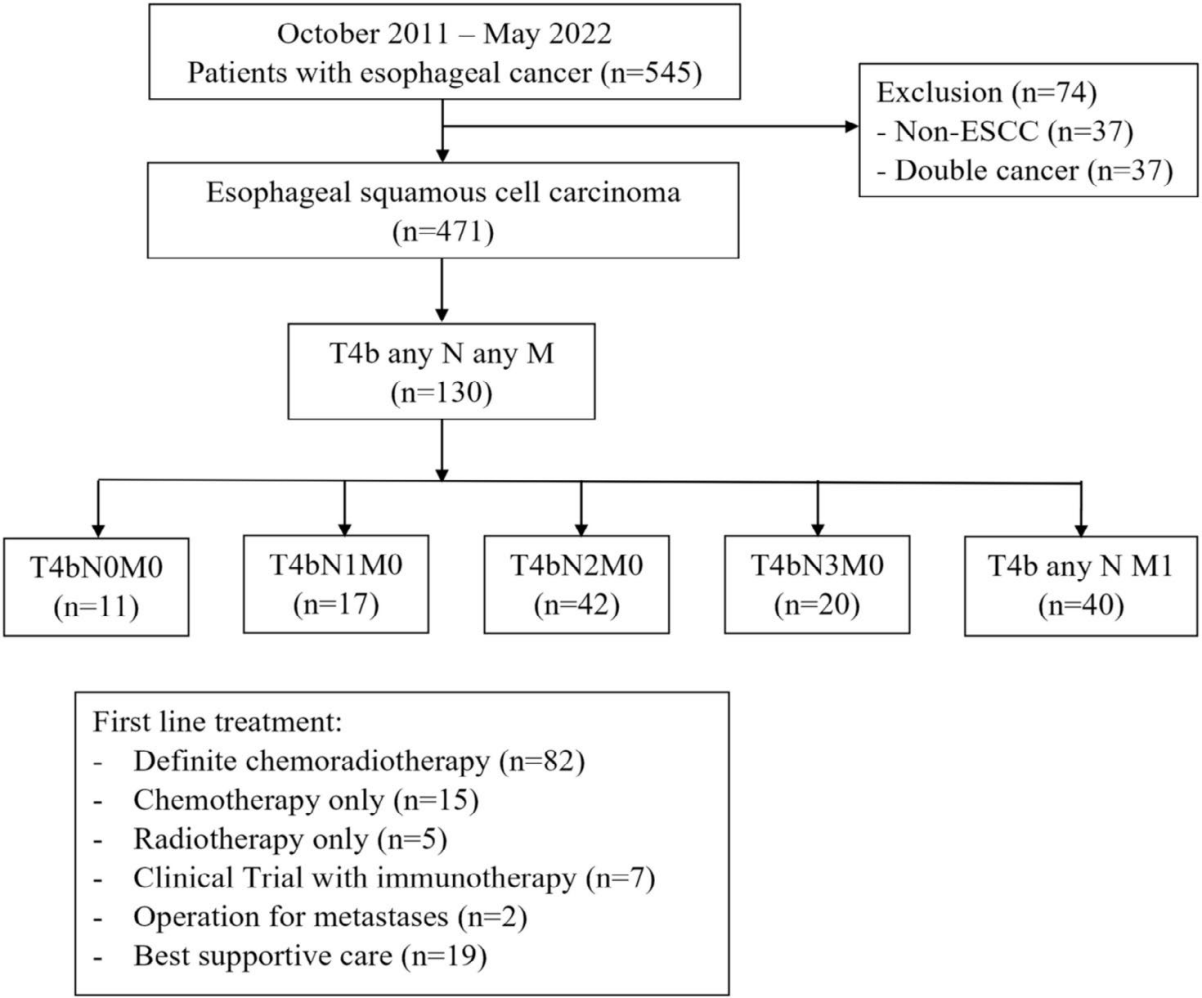
Study flowchart.

Between October 2011 and May 2022, 545 patients with esophageal cancer were diagnosed at our institute. After excluding 37 patients with non‐squamous cell carcinoma and 37 patients with synchronous double cancers, 130 out of 471 ESCC patients were classified as stage T4b and included in the final analysis. There were 11 patients in T4bN0M0, 17 patients in T4bN1M0, 42 patients in T4bN2M0, 20 patients in T4bN3M0, and 40 patients in T4bN1‐3 M1. First‐line treatment consisted of dCRT (*n* = 82), chemotherapy (C/T) alone (*n* = 15), radiotherapy (R/T) alone (*n* = 5), immunotherapy clinical trials (*n* = 7), palliative surgery for metastatic sites (*n* = 2), and best supportive care (*n* = 19). The study algorithm is demonstrated in Figure 1.

Patient outcomes were evaluated across three treatment groups: dCRT (*n* = 82), C/T alone (*n* = 15), and R/T alone (*n* = 5). The mean age was 58.2 years in the dCRT group, 61.7 years in the C/T group, and 61.3 years in the R/T group. No statistically significant differences were observed between groups regarding gender, tumor location, renal function, liver cirrhosis, body mass index (BMI) or ECOG performance status; however, patients in the C/T‐alone group exhibited a significantly higher incidence of metastasis (73.3% vs. 23.2% in the dCRT group, *p* < 0.01) and were more frequently diagnosed at an advanced clinical stage (*p* < 0.01) (Table 1).

**TABLE 1:** Demographic characteristics of T4b ESCC patients receiving dCRT, C/T, or R/T.

	dCRT (*n* = 82)	C/T (*n* = 15)	R/T (*n* = 5)	*p*
Age, years (mean ± SE)	58.2 ± 0.87	61.7 ± 2.77	61.3 ± 6.13	0.48
< 58.35	42 (51.2%)	6 (40.0%)	3 (60.0%)	0.73
≧ 58.35	40 (48.8%)	9 (60.0%)	2 (40.0%)	
Gender				> 0.99
Female	5 (6.1%)	1 (6.7%)	0 (0.0%)	
Male	77 (93.9%)	14 (93.3%)	5 (100.0%)	
N category				0.22
0	9 (11.0%)	0 (0.0%)	1 (20.0%)	
1–3	73 (89.0%)	15 (100.0%)	4 (80.0%)	
M category				< 0.01
0	63 (76.8%)	4 (26.7%)	5 (100.0%)	
1	19 (23.2%)	11 (73.3%)	0 (0.0%)	
Clinical stage				< 0.01
III	35 (42.7%)	0 (0.0%)	5 (100.0%)	
IVA	41 (50.0%)	6 (40.0%)	0 (0.0%)	
IVB	6 (7.3%)	9 (60.0%)	0 (0.0%)	
Tumor length, cm (mean ± SE)	10.2 ± 0.59	9.2 ± 0.89	7.0 ± 0.65	0.25
Baseline renal function				0.04
eGFR < 60 mL/min	16 (19.5%)	7 (46.7%)	0 (0.0%)	
eGFR ≧ 60 ml/min	66 (80.5%)	8 (53.3%)	5 (100.0%)	
Baseline BMI, kg/m^2^ (*n* = 86)				0.72
BMI < 18.5	18 (22.0%)	5 (33.3%)	1 (20.0%)	
18.5 ≦ BMI < 24	46 (56.0%)	7 (46.7%)	4 (80.0%)	
BMI ≧ 24	18 (22.0%)	3 (20.0%)	0 (0.0%)	
Baseline ECOG				0.27^a^
0	5 (6.3%)	0 (0.0%)	0 (0.0%)	
1	66 (83.6%)	10 (71.4%)	3 (75.0%)	
2	8 (10.1%)	4 (28.6%)	1 (25.0%)	
Missing	3	2	1	
Adjacent structures invasion by ESCC				0.66
Respiratory tract	73 (89.0%)	15 (100.0%)	5 (100.0%)	
Aorta	8 (9.8%)	0 (0.0%)	0 (0.0%)	
Both respiratory tract and aorta	1 (1.2%)	0 (0.0%)	0 (0.0%)	

Abbreviations: BMI, body mass index; C/T, chemotherapy; dCRT, definite chemoradiotherapy; ECOG, eastern cooperative oncology group performance status; eGFR, estimated glomerular filtration rate; ESCC, esophageal squamous cell carcinoma; R/T, radiotherapy; SE, standard error.

^a^ *P* value was calculated by excluding missing values.

**FIGURE 2:**
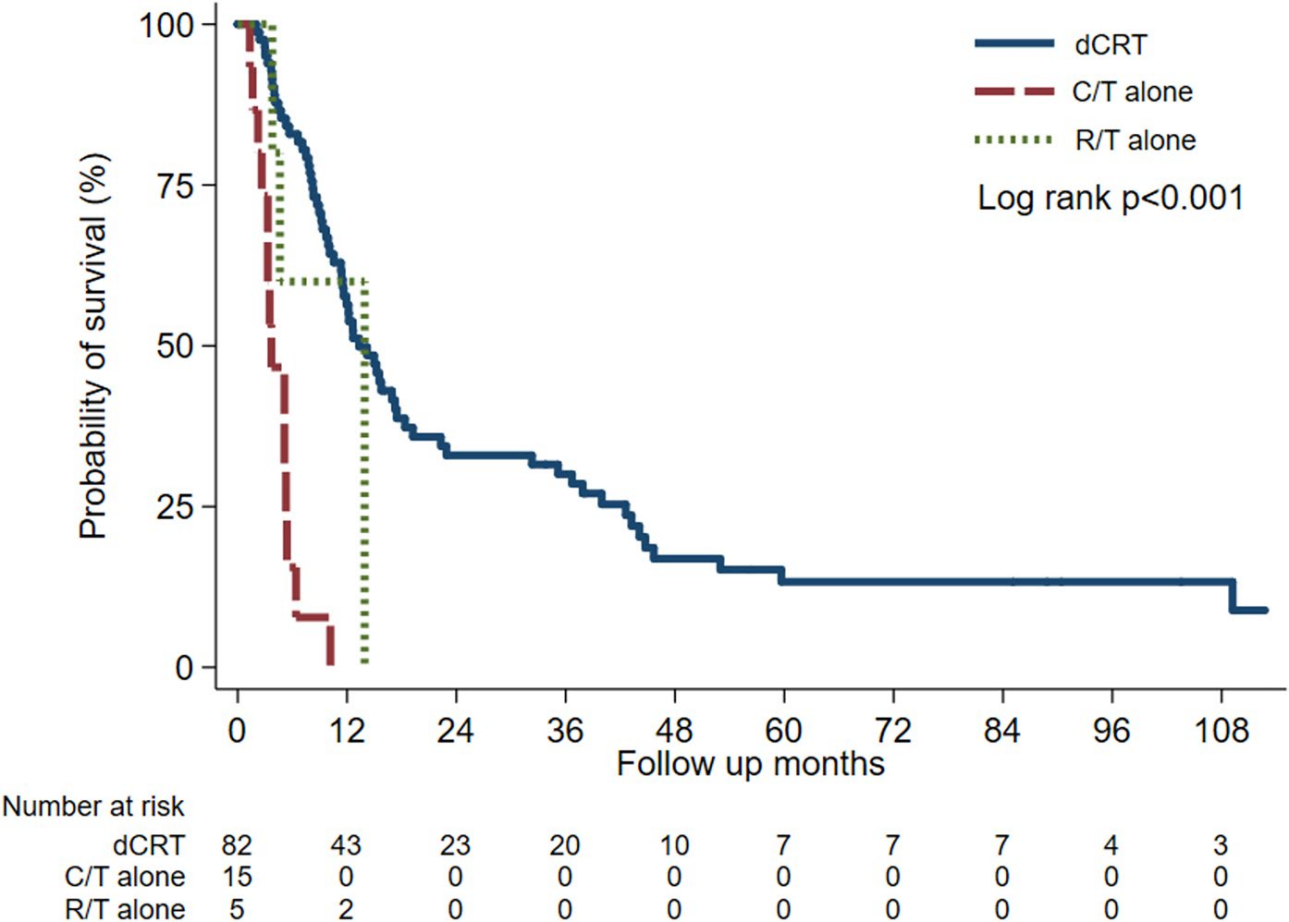
Survival in T4b ESCC patients receiving dCRT, C/T alone, or R/T alone.

Compared with monotherapy, the dCRT group demonstrated markedly improved overall survival (OS). The mean OS was 24.2 months in the dCRT group, compared with 4.2 months in the C/T group and 8.5 months in the R/T group (log‐rank test *p* < 0.001) (Figure 2). Median OS was 13.3, 3.7, and 13.9 months, respectively. In multivariable analysis adjusting for age, sex, cancer stage, treatment, esophagus fistula, and baseline ECOG, C/T alone was associated with a nearly five‐fold higher risk of death relative to dCRT (adjusted hazard ratio = 4.99, 95% CI = 2.06–12.09, *p* < 0.01) (Table 2). The estimated 1‐, 3‐, and 5‐year OS rates in the dCRT group were 56.4%, 30.0%, and 13.3%, respectively. Notably, no patient treated with either C/T or R/T survived beyond 2 years.

**TABLE 2:** Cox regression analysis of overall survival in T4b ESCC patients.

	Overall survival			
	Crude HR (95% CI)	*p*	Adjusted HR (95% CI)	*p*
Age, years (mean ± SD)	1.00 (0.98, 1.03)	0.94	0.99 (0.96, 1.02)	0.44
Gender				
Female	Ref		Ref	
Male	0.66 (0.29, 1.54)	0.34	0.97 (0.38, 2.51)	0.96
Histological grade of SCC				
1–2	Ref		—	
3	0.90 (0.53, 1.52)	0.69	—	
N category				
0	Ref		—	
1–3	1.91 (0.88, 4.15)	0.10	—	
M category				
0	Ref		—	
1	2.23 (1.39, 3.58)	< 0.01	—	
Clinical stage				
III	Ref		Ref	
IVA	2.40 (1.44, 3.99)	< 0.01	2.70 (1.52, 4.78)	< 0.01
IVB	4.79 (2.35, 9.75)	< 0.01	3.69 (1.42, 9.57)	< 0.01
Tumor location, esophagus				
Upper	Ref		—	
Middle	1.61 (1.00, 2.58)	0.049	—	
Lower	1.48 (0.78, 2.81)	0.23	—	
Baseline BMI, kg/m^2^				
BMI < 18.5	Ref		—	
18.5 ≦ BMI < 24	0.61 (0.36, 1.03)	0.07	—	
BMI ≧ 24	0.49 (0.25, 0.97)	0.04	—	
Esophagus fistula				
Without esophagus fistula	Ref		Ref	
With esophagus fistula	1.75 (1.05, 2.90)	0.03	1.38 (0.76, 2.51)	0.29
Treatment				
dCRT	Ref		Ref	
C/T alone	8.02 (4.04, 15.89)	< 0.01	4.99 (2.06, 12.09)	< 0.01
R/T alone	1.66 (0.51, 5.38)	0.40	4.32 (0.91, 20.47)	0.07
Baseline ECOG				
0	Ref		Ref	
1	0.48 (0.19, 1.21)	0.12	0.36 (0.13, 0.96)	0.04
2	0.63 (0.21, 1.84)	0.40	0.54 (0.17, 1.73)	0.30

*Note*: The cox regression model was adjusted for age, gender, clinical stage, and treatment, esophagus fistula, and baseline ECOG.

Abbreviations: BMI, body mass index; C/T, chemotherapy; dCRT, definite chemoradiotherapy; ECOG, Eastern Cooperative Oncology Group Performance Status; eGFR, estimated glomerular filtration rate; ESCC, esophageal squamous cell carcinoma; HR, hazard ratio; R/T, radiotherapy.

**FIGURE 3:**
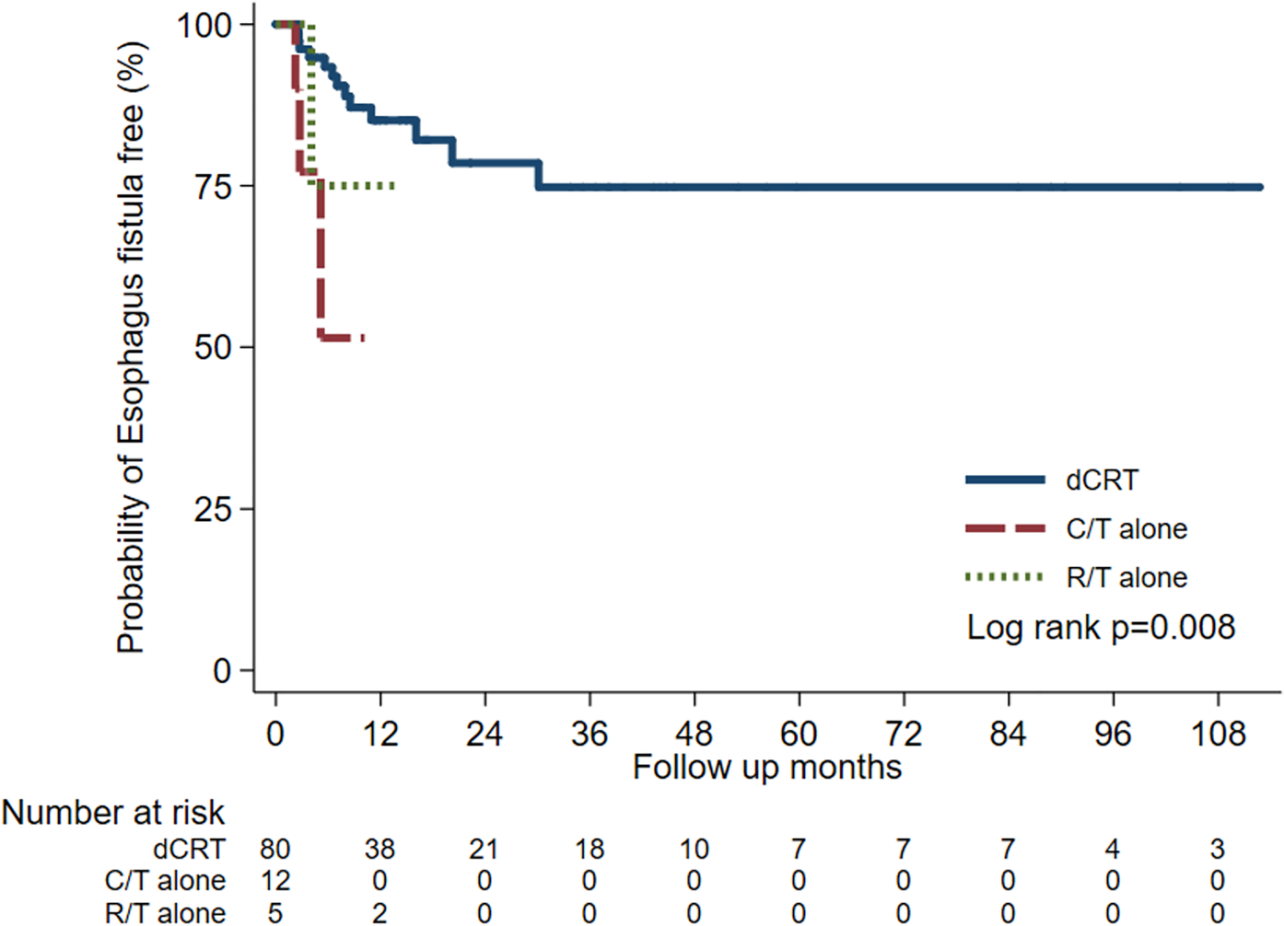
Esophagus fistula development in T4b ESCC patients receiving dCRT, C/T alone, or R/T alone.

Among 130 patients with T4b ESCC, esophageal fistula developed in 22 cases (16.9%). Of these, 19 were tracheoesophageal fistulas and three occurred at other sites, including the mediastinum and diaphragm. Among the tracheoesophageal fistulas, five were present at the time of diagnosis, three developed within 3 months of radiotherapy, and eleven developed during subsequent disease progression. The risk of esophageal fistula was significantly higher in patients treated with either C/T or R/T alone compared with those receiving dCRT (Figure 3). Furthermore, the mean time to fistula development was markedly shorter in the C/T (2.9 ± 0.7 months) and R/T (8.4 ± 2.2 months) groups than in the dCRT group (22.7 ± 3.1 months).

To identify risk factors for esophageal fistula development in T4b patients, those with fistula at initial diagnosis (*n* = 5) were excluded, leaving 17 patients with and 80 patients without esophageal fistula (Table S2). No significant differences were found between groups in age, gender, cancer stage, tumor length, tumor location, BMI, and ECOG. In univariable Cox regression, C/T alone was strongly associated with an increased risk of esophageal fistula compared with dCRT (crude HR = 6.72, 95% CI: 1.76–25.42, *p* = 0.01). This association remained significant in multivariable analysis after adjusting for age, sex, and cancer stage (adjusted HR = 11.22, 95% CI: 1.93–65.27, *p* = 0.01); but in contrast, R/T alone did not significantly increase the risk of fistula compared with dCRT (adjusted HR = 1.90, 95% CI: 0.22–16.32, *p* = 0.56). Other variables including age, gender, histological grade, nodal category, tumor length, renal function, BMI, and ECOG performance status were not significantly associated with fistula development (Table 3).

**TABLE 3:** Cox regression analysis of risk factors for esophageal fistula in T4b ESCC patients.

	Esophageal fistula			
	Crude HR (95% CI)	*p*	Adjusted HR (95% CI)	*p*
Age, years (mean ± SD)	0.96 (0.90, 1.01)	0.13	0.95 (0.89, 1.01)	0.11
Gender				
Female	Ref		Ref	
Male	0.47 (0.11, 2.09)	0.32	0.79 (0.15, 4.15)	0.78
Histological grade of SCC				
1–2	Ref		—	
3	0.94 (0.31, 2.89)	0.92	—	
N category				
0	Ref		—	
1–3	0.75 (0.21, 2.63)	0.65	—	
M category				
0	Ref		—	
1	0.84 (0.24, 2.94)	0.79	—	
Clinical stage				
III	Ref		Ref	
IVA	0.57 (0.19, 1.71)	0.32	0.56 (0.16, 1.98)	0.37
IVB	1.36 (0.29, 6.38)	0.70	0.57 (0.06, 5.31)	0.62
Tumor location, esophagus				
upper	Ref		—	
middle	1.36 (0.51, 3.62)	0.54	—	
lower	0.46 (0.06, 3.72)	0.47	—	
Tumor length, cm	0.99 (0.91, 1.09)	0.92	—	
Baseline renal function				
eGFR < 60 ml/min	0.22 (0.03, 1.69)	0.15	—	
eGFR ≧ 60 ml/min	Ref		—	
Baseline BMI, kg/m^2^				
BMI < 18.5	Ref		—	
18.5 ≦ BMI < 24	0.71 (0.24, 2.09)	0.53	—	
BMI ≧ 24	0.38 (0.07, 1.97)	0.25	—	
Treatment				
dCRT	Ref		Ref	
C/T alone	6.72 (1.76, 25.42)	0.01	11.22 (1.93, 65.27)	0.01
R/T alone	1.91 (0.24, 14.99)	0.54	1.90 (0.22, 16.23)	0.56
Baseline ECOG				
0	Ref		—	
1	0.65 (0.08, 5.07)	0.68	—	
2	0.74 (0.07, 8.26)	0.80	—	

*Note*: The cox regression model was adjusted for age, gender, clinical stage, and treatment.

Abbreviations: BMI, body mass index; C/T, chemotherapy; dCRT, definite chemoradiotherapy; ECOG, Eastern Cooperative Oncology Group Performance Status; eGFR, estimated glomerular filtration rate; ESCC, esophageal squamous cell carcinoma; HR, hazard ratio; R/T, radiotherapy.

**FIGURE 4:**
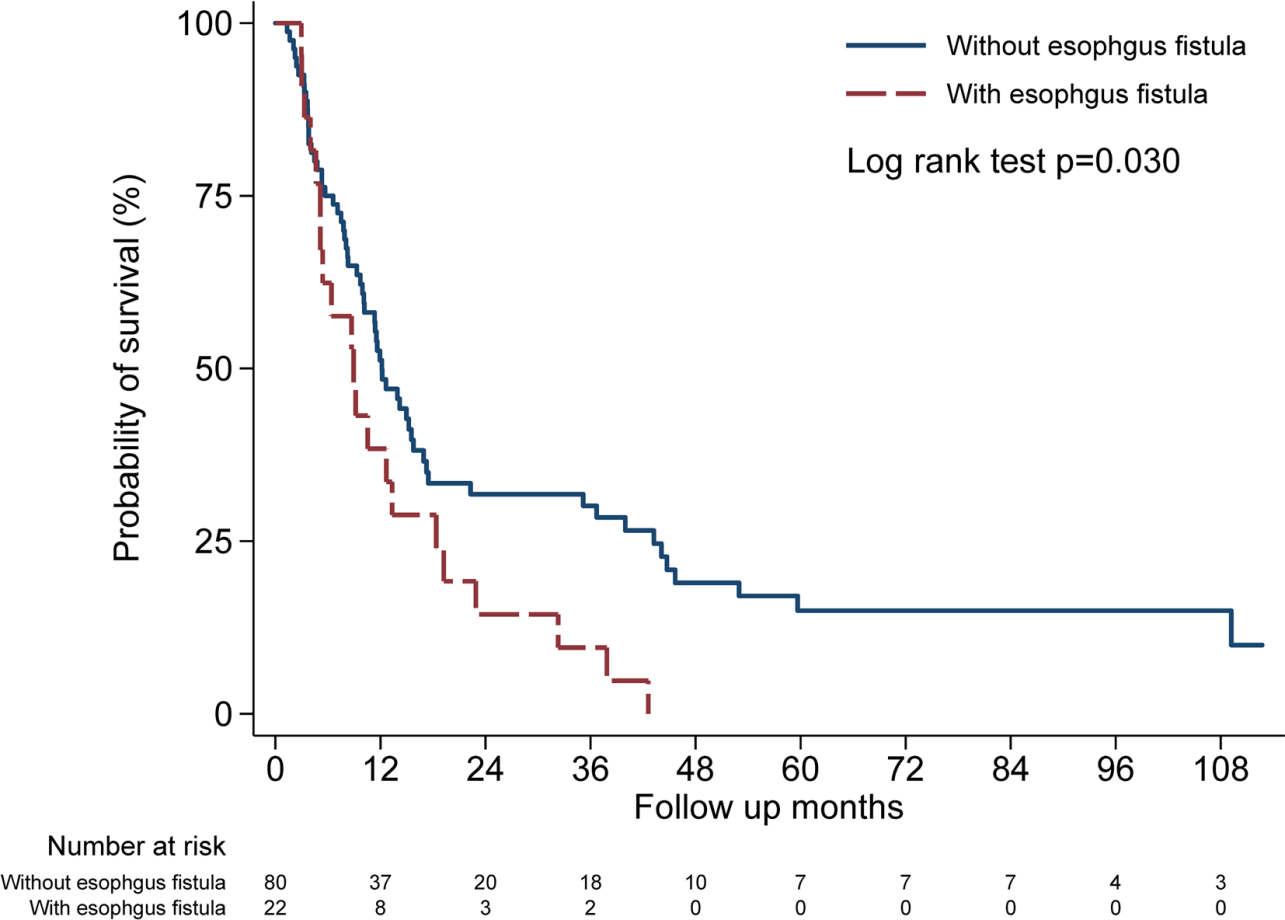
Survival in T4b ESCC patients with and without esophageal fistula.

The patients with esophageal fistulas appeared to have poorer OS than those without. The median OS for patients with and without fistula was 8.9 and 12.2 months, respectively (*p* = 0.03) (Figure 4).

## 4 Discussion

In the present study, we employed a retrospective cohort design to evaluate and compare the clinical outcomes of patients with T4b ESCC treated in the dCRT group or C/T‐ or R/T‐alone groups. Our 10‐year cohort study found that the dCRT group exhibited superior overall survival (OS) and a reduced risk of esophageal fistula development when compared to the C/T‐alone or R/T‐alone groups (Figures 2 and 3). One‐, three‐, and five‐year median OS rates in the dCRT group of our cohort were 56.4%, 30.0%, and 13.3% respectively. These findings are consistent with previous studies. A Japanese study reported a median OS of 13 months and a 2‐year OS rate of 32.1% for stage IVA ESCC (T4b or N4) [10], while a Taiwanese single‐center study reported 1‐, 3‐, and 5‐year survival rates of 49.4%, 22.2%, and 19.0% in T4b patients respectively [15]. To analyze overall survival (OS) across different cancer stages (stages III, IVA and IVB), dCRT consistently demonstrated superior OS compared to the C/T‐ or R/T‐alone groups in the subgroup analysis of our cohort (data not shown).

In our cohort, T4b ESCC patients with esophageal fistulas had poorer survival compared to those without fistulas (median overall survival: 8.9 vs. 12.2 months respectively; *p* = 0.03) (Figure 4). The mean survival of patients with and without esophageal fistulas was 12.8 and 22.6 months respectively. This result is consistent with findings from a previous single‐center study conducted in Taiwan that reported median OS in ESCC patients with and without esophageal fistulas was 10.0 and 17.2 months respectively (*p* = 0.01) [12]. Additionally, there was no significant difference in overall survival between patients with initially diagnosed esophageal fistulas and those who developed it later in our cohort (data not showed).

According to the NCCN guidelines, chemotherapy alone is recommended for T4b patients with tracheal invasion to avoid radiation‐induced esophageal fistula [13]. According to the 2022 Japanese esophageal cancer guidelines, CRT is recommended as the primary treatment modality for fit patients, as it offers a potential chance of cure despite the advanced local tumor extent [16, 17]. For patients unable to tolerate combined chemotherapy due to comorbidities such as renal dysfunction or advanced age, radiotherapy alone may be considered as an alternative; however, it is regarded as inferior in terms of tumor response and carries substantial risks, including esophageal fistula or perforation.

Importantly, there is no high‐level evidence demonstrating that radiotherapy alone improves survival in this setting and its use is largely based on clinical necessity and expert consensus rather than proven efficacy. In line with these considerations, our study provides real‐world data showing that dCRT did not significantly increase the incidence of esophageal fistula compared with C/T alone or R/T alone. A review from Japan reported esophageal fistula rates of 9%–22% in T4 ESCC patients after dCRT [18], which is consistent with our findings (17%).

In T4 ESCC patients, previous studies have identified risk factors for esophageal fistula development, such as ulcerative‐type tumors, invasion of the bronchus/trachea [8] and esophageal stenosis [11, 12]. In our study, we discovered that dCRT significantly reduced the risk of developing esophageal fistula. However, we found no associations between age, gender, cancer stage, tumor location, tumor length, BMI, ECOG performance status, or renal function and the occurrence of esophageal fistula in patients with T4b ESCC (Table 3).

In our cohort study, several factors influenced the decision for T4b patients to receive either chemotherapy or radiotherapy alone. The most prevalent reason was the patients' or their families' apprehensions regarding these treatments. Many patients expressed fear of chemotherapy due to its historically well‐known side effects and subsequently refused this option. Additionally, some physicians were concerned about the risk of esophageal fistula in cases of tracheal invasion, leading to a preference for avoiding radiotherapy. Our findings suggest that T4b patients could undergo definitive chemoradiotherapy to improve survival and potentially reduce the development of esophageal fistula. By sharing the results of our cohort, we aim to encourage more patients to undergo chemoradiotherapy, ultimately improving their prognosis.

Our study had several limitations. Firstly, despite initiating a prospective cohort study, missing data and patient loss to follow‐up introduced the potential for confounding. Secondly, the uneven distribution of patients across subgroups, with some subgroups containing a small number of cases, limited the robustness of our real‐world data analysis. To address this concern, we conducted a supplementary subgroup analysis excluding patients with metastasis (M1) (Tables S3 and S4). The results consistently demonstrated that CRT was associated with significantly improved overall survival compared with chemotherapy alone (HR = 7.7, *p* = 0.01) and radiotherapy alone (HR = 6.7, *p* = 0.03), and a significantly lower risk of fistula formation compared with chemotherapy alone (HR = 37.4, *p* < 0.01). Thirdly, we did not include patients receiving clinical trial immunotherapies due to the diversity of regimens. Finally, although viral infections such as HPV or EBV were correlated to ESCC, we did not routinely check these viral infection parameters due to health insurance policy. Further research is needed to confirm the efficacy of immunotherapy in patients with T4b ESCC.

## 5 Conclusion

Definitive chemoradiotherapy demonstrated a survival benefit for T4b ESCC patients, showing better overall survival compared to chemotherapy alone or radiotherapy alone in our cohort. Esophageal fistula was a relatively common complication in T4b ESCC patients and negatively impacted survival. Notably, chemoradiotherapy could reduce the rate of fistula formation compared to monotherapy (chemotherapy alone or radiotherapy alone).

## Conflicts of Interest

The authors declare no conflicts of interest.

## Supporting information


**Data S1:** Supporting Information.

## Data Availability

Data sharing not applicable to this article as no datasets were generated or analysed during the current study.
