# Supplementary material for: Clinical Outcomes in Patients With T4b Esophageal Squamous Cell Carcinoma: A 10‐Year Single Institution Experience
Source: Kaohsiung J Med Sci. 2025 Sep 25;42(3):e70116. doi: 10.1002/kjm2.70116 (PMC12955911; doi:10.1002/kjm2.70116)
Supplement: Supplementary file 1 — Data S1: Supporting Information. [file KJM2-42-e70116-s001.docx]

Supplementary Table 1. Demographic characteristic of T4b ESCC patients receiving dCRT, C/T alone or R/T alone

|  | dCRT (n=82) |  | C/T alone (n=15) |  | R/T alone (n=5) |  | p value |
| --- | --- | --- | --- | --- | --- | --- | --- |
| Histological grade of SCC |  |  |  |  |  |  | 0.58 ^a^ |
| 1-2 | 55 (75.34%) |  | 10 (71.43%) |  | 5 (100.00%) |  |  |
| 3 | 18 (24.66%) |  | 4 (28.57%) |  | 0 (0.00%) |  |  |
| missing | 9 |  | 1 |  | 0 |  |  |
| Tumor location, esophagus |  |  |  |  |  |  | 0.40 |
| upper | 35 (42.68%) |  | 9 (60.00%) |  | 3 (60.00%) |  |  |
| middle | 33 (40.24%) |  | 6 (40.00%) |  | 2 (40.00%) |  |  |
| lower | 14 (17.07%) |  | 0 (0.00%) |  | 0 (0.00%) |  |  |
| Tumor length, cm (mean ± SE) | 10.2 ± 0.59 |  | 9.2 ± 0.89 |  | 7.0 ± 0.65 |  | 0.25 |
| Liver cirrhosis (%) | 5 (6.10%) |  | 3 (15.00%) |  | 0 (0.00%) |  | 0.14 |
| Baseline Albumin, g/dL (mean ± SD) | 3.85 ± 0.06 |  | 3.74 ± 0.16 |  | 3.44 |  | 0.47 |
| Baseline BMI, kg/m^2^ (n=86) |  |  |  |  |  |  | 0.72 |
| BMI < 18.5 | 18 (21.95%) |  | 5 (33.33%) |  | 1 (20.00%) |  |  |
| 18.5 ≦ BMI < 24 | 46 (56.10%) |  | 7 (46.67%) |  | 4 (80.00%) |  |  |
| BMI ≧ 24 | 18 (21.95%) |  | 3 (20.00%) |  | 0 (0.00%) |  |  |
| ^a^ P value was calculated by excluding missing values.  Abbreviations: ESCC, esophageal squamous cell carcinoma; dCRT, definite chemoradiotherapy; C/T, chemotherapy; R/T, radiotherapy; eGFR, BMI, body mass index; ECOG, Eastern Cooperative Oncology Group Performance Status; SE, standard error. | | | | | | | |

Supplementary Table 2. Demographic characteristic and treatment modalities of patients with and without esophageal fistula development after diagnosis of T4b ESCC

| Variable | Esophageal fistula development  (n= 17) |  | No esophageal fistula development (n=80) |  | p value | |
| --- | --- | --- | --- | --- | --- | --- |
| Age, years (mean ± SD) | 55.44 ± 6.93 |  | 59.61 ± 8.83 |  | 0.07 | |
| < 58.35 | 10 (58.82%) |  | 39 (48.75%) |  | 0.45 | |
| ≧58.35 | 7 (41.18%) |  | 41 (51.25%) |  |  | |
| Gender |  |  |  |  | 0.28 | |
| Female | 2 (11.76%) |  | 4 (5.00%) |  |  | |
| Male | 15 (88.24%) |  | 76 (95.00%) |  |  | |
| Histological grade of SCC |  |  |  |  | >0.99 ^a^ | |
| 1-2 | 13 (76.47%) |  | 55 (76.39%) |  |  | |
| 3 | 4 (23.53%) |  | 17 (23.61%) |  |  | |
| Missing | 0 |  | 8 |  |  | |
| N category |  |  |  |  | 0.37 | |
| 0 | 3 (17.65%) |  | 7 (8.75%) |  |  | |
| 1-3 | 14 (84.35%) |  | 73 (91.25%) |  |  | |
| M category |  |  |  |  | 0.38 | |
| 0 | 14 (82.35%) |  | 55 (68.75%) |  |  | |
| 1 | 3 (17.65%) |  | 25 (31.25%) |  |  | |
| Clinical stage |  |  |  |  | 0.20 | |
| III | 10 (58.82%) |  | 28 (35.00%) |  |  | |
| IVA | 5 (29.41%) |  | 40 (50.00%) |  |  | |
| IVB | 2 (11.76%) |  | 12 (15.00%) |  |  | |
| Tumor location |  |  |  |  | 0.71 | |
| Upper | 8 (47.06%) |  | 36 (45.00%) |  |  | |
| Middle | 8 (47.06%) |  | 32 (40.00%) |  |  | |
| Lower | 1 (5.88%) |  | 12 (15.00%) |  |  | |
| Tumor length, cm | 10.00 ± 4.60 |  | 9.96 ± 5.16 |  | 0.98 | |
| Baseline renal function |  |  |  |  | 0.11 | |
| eGFR < 60 ml/min | 1 (5.88%) |  | 21 (26.25%) |  |  | |
| eGFR ≧60 ml/min | 16 (94.12%) |  | 59 (73.75%) |  |  | |
| Liver cirrhosis, yes (%) | 0 (0.00%) |  | 7 (8.75%) |  | 0.35 | |
| Baseline BMI, kg/m^2^ |  |  |  |  | 0.68 | |
| BMI < 18.5 | 5 (29.41%) |  | 19 (23.75%) |  |  | |
| 18.5 ≦ BMI < 24 | 10 (58.82%) |  | 44 (55.00%) |  |  | |
| BMI ≧ 24 | 2 (11.76%) |  | 17 (21.25%) |  |  | |
| Baseline serum Albumin, g/dL (mean ± SD) | 3.87 ± 0.53 |  | 3.84 ± 0.49 |  | 0.83 | |
| Baseline ECOG |  |  |  |  | 0.97^a^ | |
| 0 | 1 (6.25%) |  | 4 (5.19%) |  |  | |
| 1 | 13 (81.25%) |  | 62 (80.52%) |  |  | |
| 2 | 2 (12.50%) |  | 11 (14.29%) |  |  | |
| missing | 1 |  | 3 |  |  | |
| Treatment |  |  |  |  | 0.49 | |
| dCRT | 13 (76.47%) |  | 67 (83.75%) |  |  | |
| C/T or R/T only | 4 (23.53%) |  | 13 (16.25%) |  |  | |
| Treatment |  |  |  |  | 0.44 | |
| With radiotherapy | 14 (82.35%) |  | 71 (88.75%) |  |  | |
| Without radiotherapy | 3 (17.65%) |  | 9 (11.25%) |  |  | |
| Total cisplatin dose |  |  |  |  | 0.59 ^a^ | |
| ≧120mg (100%) | 10 (66.67%) |  | 39 (54.93%) |  |  | |
| 96-119mg (80%-99%) | 2 (13.33%) |  | 19 (26.76%) |  |  | |
| < 96 (<80%) | 3 (20.00%) |  | 13 (18.31%) |  |  | |
| missing | 2 |  | 9 |  |  | |
| Total 5-FU dose |  |  |  |  | 0.82 ^a^ | |
| ≧8000mg (100%) | 5 (33.33%) |  | 29 (40.85%) |  |  | |
| 6400-7999mg (80%-99%) | 4 (26.67%) |  | 15 (21.13%) |  |  | |
| < 6400 (<80%) | 6 (40.00%) |  | 27 (38.03%) |  |  | |
| missing | 2 |  | 9 |  |  | |
| Total R/T dose |  |  |  |  | 0.70 ^a^ | |
| ≧ 6000 mg | 2 (14.29%) |  | 17 (24.64%) |  |  | |
| 5000-5999 mg | 11 (78.57%) |  | 47 (68.12%) |  |  | |
| 4000-4999 mg | 0 (0.0%) |  | 2 (2.90%) |  |  | |
| < 4000 | 1 (7.14%) |  | 3 (4.35%) |  |  | |
| missing | 3 |  | 11 |  |  | |
| Descriptive variables were presented as numbers and percentages, while continuous variables were expressed as means with standard deviations. Comparisons of continuous variables were performed using Student’s t-test, and categorical variables were analyzed using Fisher's exact test.  ^a^ P value was calculated by excluding missing values.  Abbreviations: ESCC, esophageal squamous cell carcinoma; eGFR, estimated glomerular filtration rate; BMI, body mass index; ECOG, Eastern Cooperative Oncology Group Performance Status; SD, standard deviation; dCRT, definite chemoradiotherapy; C/T, chemotherapy; R/T, radiotherapy. | | | | | |  |

Supplementary Table 3. Cox regression analysis of overall survival in T4b ESCC patients without metastasis

|  | Overall Survival | | | | |
| --- | --- | --- | --- | --- | --- |
|  | Crude HR (95% CI) | p value |  | Adjusted HR (95% CI) | p value |
| Age, years (mean ± SD) | 0.99 (0.96,1.02) | 0.39 |  | 0.98 (0.95,1.02) | 0.31 |
| < 58.35 | Ref |  |  | -- |  |
| ≧58.35 | 0.85 (0.50,1.46) | 0.56 |  | -- |  |
| Gender |  |  |  |  |  |
| Female | Ref |  |  | Ref |  |
| Male | 0.83 (0.26,2.70) | 0.76 |  | 0.71 (0.20,2.47) | 0.59 |
| Histological grade of SCC |  |  |  |  |  |
| 1-2 | Ref |  |  | -- |  |
| 3 | 0.90 (0.53,1.52) | 0.69 |  | -- |  |
| N category |  |  |  |  |  |
| 0 | Ref |  |  | -- |  |
| 1-3 | 1.60 (0.72,3.55) | 0.25 |  | -- |  |
| Clinical stage |  |  |  |  |  |
| III | Ref |  |  | Ref |  |
| IVA | 2.59 (1.43,4.68) | <0.01 |  | 2.88 (1.45,5.70) | <0.01 |
| Tumor location, esophagus |  |  |  |  |  |
| upper | Ref |  |  | -- |  |
| middle | 2.03 (1.12,3.68) | 0.02 |  | -- |  |
| lower | 2.01 (0.95,4.24) | 0.07 |  | -- |  |
| Baseline BMI, kg/m^2^ |  |  |  |  |  |
| BMI < 18.5 | Ref |  |  | -- |  |
| 18.5 ≦ BMI < 24 | 0.63 (0.34,1.17) | 0.14 |  | -- |  |
| BMI ≧ 24 | 0.44 (0.19,1.01) | 0.05 |  | -- |  |
| Esophagus fistula |  |  |  |  |  |
| Without esophagus fistula | Ref |  |  | Ref |  |
| With esophagus fistula | 2.16 (1.19,3.91) | 0.01 |  | 1.41 (0.68,2.92) | 0.36 |

Supplementary Table 3. Cox regression analysis of overall survival in T4b ESCC patients without metastasis (continued)

|  | Overall Survival | | | | |
| --- | --- | --- | --- | --- | --- |
|  | Crude HR (95% CI) | p value |  | Adjusted HR (95% CI) | p value |
| Treatment |  |  |  |  |  |
| dCRT | Ref |  |  | Ref |  |
| C/T alone | 13.77 (4.06,46.66) | <0.01 |  | 7.70 (1.65,35.93) | 0.01 |
| R/T alone | 2.04 (0.62,6.75) | 0.24 |  | 6.72 (1.28,35.32) | 0.03 |
| Baseline ECOG |  |  |  |  |  |
| 0 | Ref |  |  | Ref |  |
| 1 | 0.34 (0.13,0.89) | 0.03 |  | 0.31 (0.11,0.90) | 0.03 |
| 2 | 0.37 (0.11,1.21) | 0.10 |  | 0.33 (0.09,1.25) | 0.10 |
| The cox regression model was adjusted for age, gender, clinical stage, and treatment.  Abbreviations: ESCC, esophageal squamous cell carcinoma; dCRT, definite chemoradiotherapy; C/T, chemotherapy; R/T, radiotherapy; eGFR, estimated glomerular filtration rate; BMI, body mass index; ECOG, Eastern Cooperative Oncology Group Performance Status; HR, hazard ratio. | | | | | |

Supplementary Table 4. Cox regression analysis of risk factors for esophageal fistula in T4b ESCC patients without metastasis

|  | Esophageal fistula | | | | |
| --- | --- | --- | --- | --- | --- |
|  | Crude HR (95% CI) | p value |  | Adjusted HR (95% CI) | p value |
| Age, years (mean ± SD) | 0.98 (0.92,1.05) | 0.59 |  | 0.98 (0.93,1.06) | 0.94 |
| < 58.35 | Ref |  |  | -- |  |
| ≧58.35 | 1.37 (0.48,3.91) | 0.56 |  | -- |  |
| Gender |  |  |  |  |  |
| Female | Ref |  |  | Ref |  |
| Male | 0.33 (0.07,1.49) | 0.15 |  | 0.29 (0.06, 1.37) | 0.12 |
| Histological grade of SCC |  |  |  |  |  |
| 1-2 | Ref |  |  | -- |  |
| 3 | 0.77 (0.17,3.46) | 0.73 |  | -- |  |
| N category |  |  |  |  |  |
| 0 | Ref |  |  | -- |  |
| 1-3 | 0.78 (0.22,2.82) | 0.71 |  | -- |  |
| Clinical stage |  |  |  |  |  |
| III | Ref |  |  | Ref |  |
| IVA | 0.69 (0.21,2.27) | 0.54 |  | 0.40 (0.08,1.98) | 0.26 |
| Tumor location, esophagus |  |  |  |  |  |
| upper | Ref |  |  | -- |  |
| middle | 0.78 (0.25,2.38) | 0.66 |  | -- |  |
| lower | 0.42 (0.05,3.33) | 0.41 |  | -- |  |
| Tumor length, cm | 1.01 (0.92, 1.10) | 0.87 |  | -- |  |
| Baseline renal function |  |  |  |  |  |
| eGFR < 60 ml/min | 0.31 (0.04,2.40) | 0.26 |  | -- |  |
| eGFR ≧60 ml/min | Ref |  |  | -- |  |
| Baseline BMI, kg/m^2^ |  |  |  |  |  |
| BMI < 18.5 | Ref |  |  | -- |  |
| 18.5 ≦ BMI < 24 | 1.71 (0.37,7.88) | 0.49 |  | -- |  |
| BMI ≧ 24 | 0.99 (0.14,7.06) | 0.98 |  | -- |  |
| Treatment |  |  |  |  |  |
| dCRT | Ref |  |  | Ref |  |
| C/T alone | 18.31 (3.12,107.36) | <0.01 |  | 37.38 (4.23,329.97) | <0.01 |
| R/T alone | 1.98 (0.25,15.93) | 0.52 |  | 1.72 (0.19,15.84) | 0.63 |
| Baseline ECOG |  |  |  |  |  |
| 0 | Ref |  |  | -- |  |
| 1 | 0.60 (0.07,4.84) | 0.63 |  | -- |  |
| 2 | 0.81 (0.07,9.30) | 0.87 |  | -- |  |
| The cox regression model was adjusted for age, gender, clinical stage, and treatment.  Abbreviations: ESCC, esophageal squamous cell carcinoma; dCRT, definite chemoradiotherapy; C/T, chemotherapy; R/T, radiotherapy; eGFR, estimated glomerular filtration rate; BMI, body mass index; ECOG, Eastern Cooperative Oncology Group Performance Status; HR, hazard ratio. | | | | | |
